# Nitrogen and Chemical Control Management Improve Yield and Quality in High-Density Planting of Maize by Promoting Root-Bleeding Sap and Nutrient Absorption

**DOI:** 10.3389/fpls.2022.754232

**Published:** 2022-06-23

**Authors:** Xiaoming Liu, Liguo Zhang, Yang Yu, Chunrong Qian, Congfeng Li, Shi Wei, Caifeng Li, Wanrong Gu

**Affiliations:** ^1^College of Agriculture, Northeast Agricultural University, Harbin, China; ^2^Institute of Maize Research, Heilongjiang Academy of Agricultural Sciences, Harbin, China; ^3^Institute of Crop Cultivation and Tillage, Heilongjiang Academy of Agricultural Sciences, Harbin, China; ^4^Institute of Crop Science, Chinese Academy of Agricultural Sciences, Beijing, China

**Keywords:** nitrogen fertilizer, chemical control, root bleeding sap, nutrient absorption, maize

## Abstract

High-density planting aggravates competition among plants and has a negative impact on plant growth and productivity. Nitrogen application and chemical control can improve plant growth and increase grain yield in high-density planting. Our experiment explored the effects of nitrogen fertilizer and plant growth regulators on maize root-bleeding sap, phosphorus (P) and potassium (K) accumulation and translocation, and grain yield and quality in high-density planting. We established a field study during the 2017 and 2018 growing seasons, with three nitrogen levels of N100 (100 kg ha^−1^), N200 (200 kg ha^−1^), and N300 (300 kg ha^−1^) at high-density planting (90,000 plants ha^−1^), and applied Yuhuangjin (a plant growth regulator mixture of 3% DTA-6 and 27% ethephon) at the 7th leaf. Our results showed that N200 application combined with chemical control could regulate amino acid and mineral nutrient concentration delivery rates in root-bleeding sap and improve its sap rate. Also, the treated plant exhibited higher P and K uptake and translocation ability. Furthermore, chemical control and N200 treatment maintained a high level of ribulose-1,5-bisphosphate carboxylase (RuBPCase), phosphoenolpyruvate carboxylase (PEPCase), nitrate reductase (NR), and glutamine synthetase (GS) enzymatic activities in leaves. In addition, plant growth regulator and nitrogen application improved the enzymatic activities of GS, glutamate dehydrogenase (GDH), and glutamic pyruvic transaminase (GPT) and the contents of crude protein, lysine, sucrose, and soluble sugar in grain and ultimately increased maize yield. This study suggests that N200 application in combination with chemical control promotes root vitality and nutrient accumulation and could improve grain yield and quality in high-density planting.

## Introduction

The root is an essential absorption system, and its function is to maintain the supply of nutrients and soil moisture for crop growth and development (Xu et al., 2009; Fan et al., 2021). The root system of crops greatly influences the above-ground growth and biomass yield, which play an important role in yield formation (Yang et al., 2004; Chen et al., 2022). The capacity for nutrient and soil moisture uptake by crops is directly influenced by root development and root activity strength (Li et al., 2019). Well-developed root systems are always accompanied by vigorous above-ground growth and high yields. Root-bleeding sap is a sign of root pressure, and its change is consistent with root activity (Xu et al., 2016). The root-bleeding sap is directly correlated to the uptake of nutrients and water and reflects the root system's potential for plant growth and root activity (Ansari et al., 2004; Noguchi et al., 2005). The concentration of nutrients in root-bleeding sap represents the nutritional status and reflects root absorption and translocation rates in crops (Noguchi et al., 2005; Nishanth and Biswas, 2008). Hence, an appropriate rate of root-bleeding sap is vital to optimizing maize yield and directly influencing maize growth and development.

Nutrient absorption and translocation in crops are the physiological basis for dry matter accumulation and yield formation, influencing crop growth and development (Wu et al., 2018; Li et al., 2021). The difference in biomass yield is closely correlated to the plant's nutrient uptake and utilization characteristics. It is generally believed that obtaining a higher yield requires crops to absorb a large amount of nutrients from the soil (Wu et al., 2015; Zhan et al., 2016). Phosphorus promotes carbohydrate and starch synthesis in stems and leaves and increases the nutrient transport to the grains, thereby improving grain weight and quality (Wang and Ning, 2019). Potassium can stimulate the synthesis and transport of carbohydrates and promote the growth of maize ear (Shahzad et al., 2017). Phosphorus and potassium are nutrient elements in great demand for maize. Adequate P and K supply promotes root development and dry matter accumulation and enhances maize's resistance to stress (Xie et al., 2011; Iqbal et al., 2020). Furthermore, maize's adequate P and K contents promote the grain development process and help in obtaining a relatively high grain number per ear and weight (Liu et al., 2011). Therefore, the absorption and translocation of P and K play an important role in maize growth and yield potential in the process of yield formation.

Maize (*Zea mays* L.) is one of the most essential cereal feeds worldwide and occupies a prominent place in global food security and sustainable development (Palacios-Rojas et al., 2020). Since the mid-1990s, with the improvement of the economy and dietary structure in China, the consumption of animal-derived foods, such as meat, milk, and eggs, has increased, which rapidly increased the demand for maize. Maize is the most widely cultivated crop in China, and its production reflects people's need (Liu S. Q. et al., 2021). Northeast China is a major maize producing region, and its planting area and yield account for 31 and 34%, respectively, of the total maize production in China (Liu and Ye, 2020). The current maize planting density in Northeast China is relatively low, resulting in fewer grain yields (Luo et al., 2020). Maize yield in this region has only reached 50% of its yield potential, which offers an excellent opportunity for increasing yield. It is generally accepted that relying on high-density planting to enhance population productivity is one of the most important measures to increase yield potential (Tang et al., 2018). However, high-density planting increases resource competition among maize plants, leading to a decline in individual plant productivity and negatively affecting yield potential (Rossini et al., 2011). This inevitably intensifies the competition betwen the root systems as it is an important organ for maize to obtain environmental resources. Increased planting density leads to decreased row spacing, resulting in increased nutrients, water, and space competition between maize plants. It also severely limits the spatial distribution of the root system and restricts the capacity of nutrient absorption and utilization, ultimately leading to a decline in root quality and grain yield (Gao et al., 2021). According to Shao et al. (2018), root length and root number per plant decrease significantly as planting density increases. The increase in planting density not only inhibits the growth, quantity, and quality of maize roots but also reduces nutrient absorption and translocation in maize (Li et al., 2020; Gao et al., 2021). Therefore, enhancing root physiological characteristics and nutrient absorption capacity in high-density planting for optimal maize growth and high yield has become a significant problem in maize production.

A sufficient supply of nutrients has become essential to achieving high crop yield under high-density planting. Nitrogen, one of the most critical nutrient elements during the maize growing period, greatly affects the root morphological characteristics and physiological activities (Li et al., 2019). It is reported that nitrogen application could significantly increase the total length, volume, and effective absorption area of roots, thereby improving root nutrient absorption capacity (Liu et al., 2017). Furthermore, nitrogen fertilizer plays an important role in the crop's nutrient accumulation and transport activity. Appropriate nitrogen application can increase the grain yield by increasing nutrient accumulation post-anthesis and nutrient translocation to grains (Zhang et al., 2021). Chemical control is one of the efficient cultivation measures, which regulates plant growth and development process, enhances nutrient utilization capacity and environment adaptability, and improves grain yield and quality (Hutsch and Schubert, 2017; Stutts et al., 2018). The application of plant growth regulators can enhance the capacity of crops to absorb nutrients and soil moisture by improving their root growth characteristics (Lin et al., 2019; Nawaz et al., 2020). Yuhuangjin is a type of plant growth regulator that is widely used in maize production in China. The main component is ethephon and diethyl aminoethyl hexanoate DTA-6, which improves plant growth, enhances lodging resistance, optimizes yield component, and increases yield (Zhang et al., 2014). Therefore, we hypothesized that chemical control and nitrogen fertilizer could improve root growth, increase nutrient absorption, and promote yield formation in maize. To prove this hypothesis, this study investigated the effects of chemical control and nitrogen fertilizers on root-bleeding sap characteristics, P and K accumulation and translocation, and grain yield and quality in high plant density. This study aimed to provide a theoretical basis for increasing maize yield and quality in future high-density planting management practices.

## Materials and Methods

### Site Description

**Figure 1:**
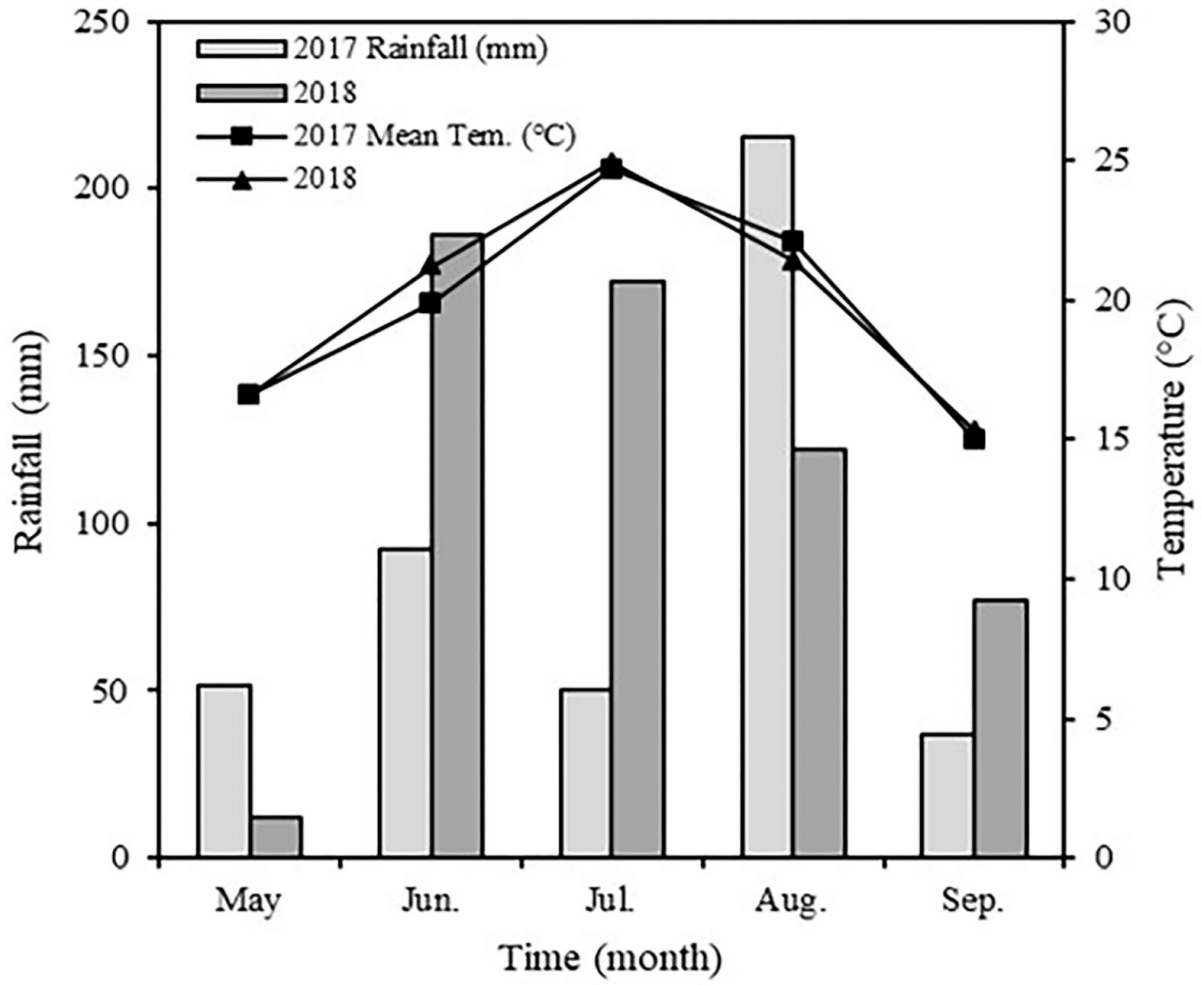
Monthly rainfall distribution and mean temperature during spring maize growing stage in 2017 and 2018.

The experiment was conducted from April to September in 2017 and 2018 at the experimental station of Northeast Agricultural University, Harbin, Heilongjiang Province, China (126°54′E, 45°46′N). The region has a typical warm temperate monsoon climate with an annual mean temperature of 4.5°C and annual mean precipitation of 569 mm. The crop rotation system is continuous maize cropping, and the soil type at the experimental site is chernozem. The physical and chemical characteristics of tillage layer soil were pH 6.85; organic matter 25.25 g kg^−1^; total nitrogen 1.70 g kg^−1^; available phosphorus 65.34 mg kg^−1^; and available potassium 179.35 mg kg^−1^. Temperature and rainfall during the growth stage of spring maize in 2017 and 2018 are shown in Figure 1.

### Experimental Design and Field Management

The experiment was laid out as a split-plot design with three replicates. Two chemical treatments (Y, Yuhuangjin; Control (CK), water) were used as the main plots, and three nitrogen fertilizer levels were used as the subplots: 100 kg ha^−1^ (N100), 200 kg ha^−1^ (N200), and 300 kg ha^−1^ (N300). The plant growth regulator Yuhuangjin (the mixture of 3% DTA-6 and 27% ethephon) was provided by Haolun Co., Ltd., Fujian, China. About 0.83 mL L^−1^ of Yuhuangjin solution was sprayed on the foliar surface at the seven-leaf stage in the afternoons between 16:00 and 18:00 h. Yuhuangjin was applied at 450 L ha^−1^, and the same volume of water was applied to the control plants. Spring maize Longyu 365, a high-yielding variety in Heilongjiang province, was sown manually at 90,000 plants ha^−1^ on 30 April and harvested on 25 September in 2017 and 2018. The size of each plot was 5.2 × 8 m with 0.65 m row spacing. All plots were supplied with 100 kg ha^−1^ P_2_O_5_ and 100 kg ha^−1^ K_2_O. The total phosphorus and potassium and half of the nitrogen (urea, 46% N) were applied at the sowing. The balance half of the nitrogen was applied at the jointing stage. No irrigation was applied during the maize growing season. Pests, weeds, and diseases were controlled in a timely manner, and tillage management was conducted according to local farmer management.

### Collection of Root-Bleeding Sap

Three representative plants were sampled from each plot at jointing, tasseling, early grain filling, and milking stages. The plants were cut at the third basal internode using lopping shears at 19:00 h. The incision was washed with distilled water, covered with a centrifuge tube containing degreasing cotton (≈2/3 of the centrifugal tube volume), and secured with plastic wrap to collect the root-bleeding sap. The centrifuge tubes were collected at 6:00 h the next day, and the weight was measured (Wang H. et al., 2019). The bleeding sap rate was calculated as the weight increase of the centrifuge tube per hour per plant (g h^−1^ plant^−1^).

### Analysis of Root-Bleeding Sap Components

Concentrations of serine (Ser), glutamic acid (Glu), glycine (Gly), alanine (Ala), valine (Val), lysine (Lys), methionine (Met), arginine (Arg), and leucine (Leu) in the root-bleeding sap were measured using high-performance liquid chromatography with pre-column derivatization (Li H. W. et al., 2012). Concentrations of P, K, Ca, Mg, Fe and Zn were measured using inductively coupled plasma optical emission spectroscopy (ICP-AES, OPTIMA 3300 DV, Perkin-Elmer, USA).

### Determination of Photosynthesis and N Metabolism Enzyme Activities in Ear Leaf

Approximately 0.5 g of fresh ear leaf was homogenized with an extraction medium (pH 8.4, 0.1 mmol L^−1^ Tricine-HCl, 10 mmol L^−1^ MgCl_2_, 1 mmol L^−1^ EDTA, 7 mmol L^−1^ β-mercaptoethanol, 5% glycerol (v/v) and 1% PVP) in an ice-cold mortar with a pestle. The homogenate was centrifuged at 15,000 × g for 10 min at 4°C. The supernatant was used for the RuBPCase and PEPCase assays following the methods of Lilley and Walker (1974) and Arnozis et al. (1988), respectively.

Approximately 1 g of fresh ear leaf was homogenized with the extraction medium (pH 7.5, 0.1 mol L^−1^ Tris-NaOH, 5 mmol L^−1^ MgCl_2_ and 1 mmol L^−1^ DTT) precooled in ice, followed by centrifugation at 20,000 × g for 15 min at 4°C. The supernatant was used for enzyme assays. Nitrate reductase (NR) activity was determined by the method of Lewis et al. (1982), and glutamine synthetase (GS) activity was determined by the method of Canovas et al. (1991).

### Determination of N Metabolism Enzyme Activity in Grain

Three ears per plot were randomly sampled at 10, 15, 20, 25, and 30 days after silking. Approximately 100 grains in the middle of the ear were collected and frozen in liquid N_2_ and stored at −80°C for enzyme assays. About 0.5 g of frozen grain was homogenized with phosphate buffer (pH 7.2), followed by centrifugation at 10,000 × g for 20 min. The supernatant was used for enzyme assays of GS and glutamate dehydrogenase (NADH-GDH and NAD-GDH) activities following the method of Wang et al. (2016).

About 0.2 g of frozen grain was homogenized with Tris-HCl extraction buffer (pH 7.2, 50 mmol L^−1^ trihydroxymethyl aminomethane) precooled in ice, followed by centrifugation at 20,000 × g for 20 min at 4°C. The supernatant was used for the glutamic-pyruvic transaminase (GPT) assay following the method of Wang et al. (2016).

### Analysis of Nutrients Concentration in Grain

The grains were sampled and oven-dried at 40°C for 24 h and ground to powder at harvest. The resulting grain powder was passed through a 0.25 mm mesh and stored at 4°C for analysis. Crude protein in grain was assayed by the micro-Kjeldahl method described by the Association of Official Agricultural Chemists AOAC (1975). Crude fat was assayed following the method of AOAC (1984). Starch was assayed by the colorimetric method described by Boros et al. (2004). Lysine was assayed using the colorimetric method described by Reddy et al. (2013).

Approximately 1 g of fresh grain was ground in a mortar with liquid nitrogen, and 10 ml of distilled water was added to the sample and incubated in boiling water for 60 min. The mixture was centrifuged at 12,000 × g for 20 min at 4°C. The supernatant was used for soluble sugar and sucrose measures. Soluble sugar was measured by the anthrone colorimetric method described by Liu et al. (2007). Sucrose was measured by the anthrone method described by Van (1968).

### Determination of P and K Accumulation and Translocation

Three plants were sampled from each plot and separated into stems, leaves, and grains during harvest. The samples were dried in an oven at 105°C for 30 min and afterward at 80°C to a constant weight. Dried samples were weighed and ground to pass through a 1-mm sieve and digested by an H_2_SO_4_-H_2_O_2_ mixture (Wolf, 1982). The P concentration was determined by the ammonium molybdate ascorbic acid reduction method (Murphy and Riley, 1962). The K concentration was determined by the flame photometer method. Nutrient (P or K) accumulation was calculated based on the sum of the dry matter and P or K concentration in plant parts.

Nutrient (P or K) translocation amount of pre-silking (TAE, kg ha^−1^) = vegetative organ nutrient (P or K) content at silking—vegetative organ nutrient (P or K) content at maturity.

Nutrient (P or K) translocation rate of pre-silking (TRE, %) = TAE/vegetative organ nutrient (P or K) content at silking × 100.

Contribution rate of nutrient (P or K) translocation amount of pre-silking (CTAE, %) = TAE/grain nutrient (P or K) content at maturity × 100.

Nutrient (P or K) accumulation amount of post-silking (AAT, kg ha^−1^) = plant nutrient (P or K) content at maturity – plant nutrient (P or K) content at silking.

Contribution rate of nutrient (P or K) accumulation amount of post-silking (CAAT, %) = AAT/grain nutrient (P or K) content at maturity × 100.

### Statistical Analysis

The data were summarized to calculate the mean value and standard error (SE). The mean value was compared by the analysis of variance (ANOVA) to analyze the significant differences between samples with different treatments (*P* < 0.05). All statistical analyses were performed by SPSS 19.0 procedures (SPSS Inc., Chicago, IL, USA). Microsoft Excel 2010 was used to draw tables.

## Results

### Root-Bleeding Sap and Nutrients Composition Delivery Rate

The chemical control and nitrogen fertilization exhibited a significant influence on the rate of root-bleeding sap during the maize growing period in 2017 and 2018 (Table 1). At the same N levels, chemical control increased root-bleeding sap rate with an average augment of 12.26, 15.99, 14.21, 8.97, and 18.46% from the jointing stage to the maturing stage compared with water treatment. Root-bleeding sap rate first increased and then decreased with the increase of nitrogen application under the same chemical treatment, and the highest value was measured under N200 treatment. The results show that a high N level inhibited the increase of root-bleeding. An analysis of the synthetic effect revealed that the highest root-bleeding sap rate was obtained from N200 application under chemical control.

**Table 1:** Effects of chemical control and nitrogen fertilizers on root-bleeding sap rate (μg h^−1^ plant^−1^) during the maize growing period in 2017 and 2018.

**Year**	**Treatment**	**Jointing stage**	**Tasseling stage**	**Early filling stage**	**Milk stage**	**Maturing stage**
2017	N100+CK	1.42d	1.75d	1.99d	2.75cd	0.76d
	N200+CK	1.56c	1.96c	2.17c	2.79c	0.86c
	N300+CK	1.47d	1.85cd	2.08cd	2.71d	0.82c
	N100+Y	1.58c	2.07b	2.31b	2.99b	0.92b
	N200+Y	1.77a	2.26a	2.49a	3.10a	1.03a
	N300+Y	1.67b	2.20a	2.38b	2.98b	0.98a
2018	N100+CK	1.36c	1.65c	2.03d	2.50c	0.70c
	N200+CK	1.49b	1.87b	2.24bc	2.64b	0.80b
	N300+CK	1.47b	1.75c	2.15c	2.56c	0.77b
	N100+Y	1.53b	1.95b	2.32b	2.70b	0.82b
	N200+Y	1.67a	2.10a	2.50a	2.85a	0.91a
	N300+Y	1.61ab	1.98b	2.45a	2.77ab	0.90a

*N100+CK, N200+CK, and N300+CK indicate nitrogen applied levels at 100, 200, and 300 kg ha^−1^ under water treatment, respectively; N100+Y, N200+Y, and N300+Y indicate nitrogen applied levels at 100, 200, and 300 kg ha^−1^ under chemical control, respectively. Means within a column for the same year followed by the different letters indicate a significant difference at P < 0.05*.

The delivery rate of free amino acids in root-bleeding sap was influenced by chemical control and nitrogen fertilizer, which decreased after the jointing stage in maize (Table 2). At the same N levels, chemical control increased the delivery rate of Ser, Glu, Gly, Ala, Val, Lys, Met, Arg, and Leu with an average augment of ≈11.45–19.04% than water treatment at the tasseling stage in both years, which was consistent at different growth stages. Under the same chemical treatment, the free amino acid delivery rate obtained the highest value under N200 treatment, which showed an average augment of 6.54–15.04% and of 4.15–6.97% compared with N100 and N300 nitrogen rates in both years. From the analysis of synthetic effect, the delivery rate of free amino acids in root-bleeding sap was optimal in N200 application under chemical control.

**Table 2:** Effects of chemical control and nitrogen fertilizers on amino acids concentrations (μg h^−1^ plant^−1^) in root-bleeding sap during the maize growing period in 2017 and 2018.

**Growth stage**	**Treatment**	**2017**									**2018**								
		**Ser**	**Glu**	**Gly**	**Ala**	**Val**	**Lys**	**Met**	**Arg**	**Leu**	**Ser**	**Glu**	**Gly**	**Ala**	**Val**	**Lys**	**Met**	**Arg**	**Leu**
Jointing stage	N100+CK	479.35d	284.97d	1.34d	13.91d	57.59d	92.92e	5.46c	85.15d	17.88e	468.15d	279.68d	1.27d	13.39d	54.58d	89.06d	4.95d	83.29d	16.63d
	N200+CK	506.23c	310.99c	1.44c	15.38c	62.68bc	101.63cd	5.87b	95.36b	19.47cd	495.77bc	303.61c	1.38c	14.56bc	59.63bc	96.43c	5.26c	90.14c	18.15c
	N300+CK	496.70cd	307.06c	1.40cd	14.26d	59.52cd	96.61de	5.30c	89.93c	18.36de	482.89cd	295.27c	1.31d	14.07c	57.71c	91.45d	5.13cd	87.15cd	17.36cd
	N100+Y	539.22b	334.64b	1.56b	16.01bc	66.32b	105.60bc	6.00b	99.17b	20.86bc	516.74b	327.46b	1.45b	15.22b	62.46b	101.54b	5.59b	94.82b	19.43b
	N200+Y	568.89a	359.67a	1.65a	17.53a	71.86a	116.71a	6.48a	105.17a	22.92a	543.23a	346.84a	1.57a	16.24a	66.87a	109.18a	6.11a	100.48a	21.32a
	N300+Y	544.89ab	339.95b	1.56b	16.51ab	67.04ab	108.43b	6.17ab	99.18b	21.74ab	525.28ab	335.29ab	1.49b	15.48ab	63.52b	104.77ab	5.78b	96.57ab	20.08b
Tasseling stage	N100+CK	377.14d	227.46d	1.13d	11.69d	49.83d	73.17d	4.37d	74.47e	13.91c	365.26d	212.76d	1.04d	11.24d	45.62d	70.33d	4.05d	69.02d	13.34d
	N200+CK	403.18c	242.68c	1.22c	12.64c	53.64cd	82.09c	4.72c	80.27cd	15.97b	386.53c	230.53c	1.12c	12.29c	50.57c	78.05c	4.48bc	75.24bc	15.36b
	N300+CK	383.42d	226.22d	1.13d	11.74d	49.96d	73.17d	4.28d	76.75de	14.39c	370.72cd	218.42d	1.07cd	11.73cd	47.45d	72.48d	4.29c	72.65c	14.21c
	N100+Y	424.42b	255.84b	1.26bc	13.55b	55.78bc	86.38b	5.07b	83.34bc	17.40a	409.58b	245.84b	1.19b	13.26b	52.63c	82.09b	4.66b	78.73b	16.02b
	N200+Y	453.42a	278.94a	1.36a	14.70a	61.24a	94.45a	5.33a	89.53a	18.28a	435.21a	264.39a	1.30a	14.02a	59.29a	88.84a	5.03a	85.82a	17.27a
	N300+Y	438.67ab	265.77b	1.31ab	14.05ab	58.21ab	89.34b	4.97b	85.13b	17.02ab	422.47ab	254.56ab	1.23b	13.68ab	56.34b	84.27b	4.83ab	80.56b	16.29b
Early filling stage	N100+CK	318.24c	173.80c	0.71c	9.88e	44.29c	61.05e	3.99d	66.60c	7.71c	302.85c	169.82d	0.69d	9.43d	40.03d	60.61d	3.68d	62.14d	7.06d
	N200+CK	342.15bc	197.32b	0.77b	11.05cd	48.62b	69.74c	4.31c	72.01b	9.14b	333.52b	185.35c	0.74c	10.24c	44.67c	66.44c	4.04bc	70.47b	8.23c
	N300+CK	329.59bc	184.54c	0.71c	10.36de	44.05c	65.30d	4.01d	66.90c	7.75c	315.62c	177.47cd	0.70d	9.77cd	42.98c	63.25d	3.89c	66.44c	7.32d
	N100+Y	367.79ab	209.01b	0.88a	11.59bc	51.53a	72.88c	4.42bc	74.67b	9.47b	345.07b	198.09b	0.83b	10.88b	47.44b	72.96b	4.21b	73.92ab	8.98b
	N200+Y	383.05a	230.34a	0.90a	12.86a	54.37a	83.57a	4.90a	79.70a	10.80a	367.26a	221.53a	0.88a	12.02a	51.85a	78.37a	4.65a	77.25a	9.75a
	N300+Y	365.08ab	222.42a	0.87a	12.25ab	52.57a	77.77b	4.61b	78.99a	9.84ab	351.63ab	207.04b	0.85ab	11.34b	48.62b	76.72a	4.36b	75.34a	9.52a
Milk stage	N100+CK	147.14d	108.01e	0.50e	6.00d	23.39d	39.69d	2.02cd	29.19c	3.86c	148.53d	110.84d	0.55e	5.89d	22.08e	39.82d	1.78d	27.14e	4.14e
	N200+CK	169.19c	123.96cd	0.69c	7.08c	29.31b	47.88c	2.17c	36.59b	4.99b	163.29c	120.06c	0.64c	6.78c	25.31c	45.47c	1.92c	32.19c	5.05c
	N300+CK	162.04c	119.80de	0.63d	6.68c	26.81c	44.55c	1.95d	31.15c	4.25c	157.24c	114.26d	0.59d	6.14d	23.86d	41.19d	1.83cd	30.18d	4.63d
	N100+Y	183.75b	134.16bc	0.71bc	8.04b	30.24b	52.28b	2.51b	39.02b	5.33b	179.08b	130.32b	0.70b	7.66b	28.75b	50.95b	2.27b	34.63b	5.51b
	N200+Y	209.35a	148.63a	0.82a	8.89a	33.84a	60.34a	2.77a	45.58a	6.46a	192.41a	142.89a	0.76a	8.25a	31.87a	56.21a	2.49a	40.52a	6.11a
	N300+Y	190.15b	144.44ab	0.75b	8.14b	32.59a	55.58b	2.46b	39.37b	6.07a	184.47ab	136.93a	0.72b	7.62b	29.24b	53.02b	2.35b	35.79b	5.78b

*N100+CK, N200+CK, and N300+CK indicate nitrogen applied levels at 100, 200, and 300 kg ha^−1^ under water treatment, respectively; N100+Y, N200+Y, and N300+Y indicate nitrogen applied levels at 100, 200, and 300 kg ha^−1^ under chemical control, respectively. Means within a column for the same growth stage followed by the different letters indicate a significant difference at P < 0.05*.

A similar change trend was observed in the mineral nutrient concentrations in bleeding sap during the maize growing period in 2017 and 2018 (Table 3). The mineral nutrient concentrations were significantly affected by chemical control and nitrogen fertilizer. The delivery rate of mineral nutrients first increased and then decreased with the increase of nitrogen application under the same chemical treatment. At the same N levels, chemical control obviously increased the delivery rate of mineral nutrients at different growth stages. From the analysis of synthetic effect, the delivery rate of mineral nutrients in root-bleeding sap was optimal in N200 application under chemical control.

**Table 3:** Effects of chemical control and nitrogen fertilizers on mineral nutrients concentrations (μg h^−1^ plant^−1^) in root-bleeding sap during the maize growing period in 2017 and 2018.

**Growth period**	**Treatment**	**2017**											**2018**										
		**Fe**	**Mn**	**Cu**	**Zn**	**Ca**	**Mg**	**Mo**	**K**	**P**	**B**	**Si**	**Fe**	**Mn**	**Cu**	**Zn**	**Ca**	**Mg**	**Mo**	**K**	**P**	**B**	**Si**
Jointing stage	N100+CK	1.62d	4.37d	0.046c	10.93c	318.23c	299.46cd	0.057d	1747.53e	113.76c	1.14cd	50.45c	1.55d	4.15d	0.043d	10.08d	302.98d	259.46d	0.051d	1682.49d	104.07d	1.01d	44.37d
	N200+CK	1.80c	4.75c	0.050bc	11.55bc	347.74b	314.76bc	0.064c	1920.50cd	123.26bc	1.21c	53.66b	1.79c	4.62c	0.049c	11.39bc	332.63bc	290.56c	0.058c	1801.37c	115.48c	1.11c	48.62c
	N300+CK	1.87c	4.80c	0.051bc	11.66b	339.26b	284.37d	0.055d	1833.47de	118.52c	1.07d	53.96b	1.77c	4.51c	0.047c	10.92c	320.06c	278.85c	0.056c	1754.88cd	110.75c	1.04d	45.89d
	N100+Y	2.06b	5.22b	0.056ab	12.43a	350.32b	332.33ab	0.070b	1998.30bc	134.01ab	1.37b	55.22b	1.96b	5.07b	0.053b	11.85b	346.45b	307.82b	0.064b	1895.03b	122.62b	1.26b	51.84b
	N200+Y	2.17a	5.50ab	0.060a	12.82a	383.02a	345.53a	0.075a	2124.70a	142.78a	1.49a	58.39a	2.12a	5.43a	0.058a	12.77a	370.82a	333.13a	0.071a	2093.27a	136.59a	1.39a	56.25a
	N300+Y	2.20a	5.57a	0.062a	13.05a	374.64a	328.45ab	0.068b	2087.37ab	138.60a	1.37b	58.97a	2.08a	5.39a	0.057a	12.64a	362.17ab	316.28b	0.066b	1956.36b	127.94b	1.28b	53.08b
Tasseling stage	N100+CK	0.49d	4.29d	0.063e	9.26d	300.46e	292.80d	0.062d	1538.44d	110.49c	1.09d	44.41d	0.52e	4.05d	0.58d	8.98d	282.94d	267.25d	0.059d	1496.05d	98.72c	1.00d	42.67d
	N200+CK	0.65c	4.73c	0.069d	9.97c	320.38cd	310.57bc	0.075c	1671.27c	118.37bc	1.18c	46.93c	0.62c	4.52bc	0.64c	9.53c	303.39c	285.03bc	0.069c	1602.88c	109.46b	1.10c	45.42c
	N300+CK	0.71c	4.90c	0.071cd	9.92c	307.73de	297.60cd	0.071c	1608.97cd	110.54c	1.09d	45.82cd	0.58d	4.37c	0.62c	9.39cd	287.33d	276.29cd	0.066c	1579.14c	102.78c	1.06c	44.68cd
	N100+Y	0.77b	5.04bc	0.074bc	10.93b	344.16ab	328.07a	0.086b	1760.22b	123.53b	1.28b	49.99b	0.74b	4.68b	0.69b	10.21b	320.17b	297.42b	0.077b	1693.49b	112.53b	1.17b	48.39b
	N200+Y	0.90a	5.40ab	0.078ab	11.48a	359.44a	335.49a	0.094a	1874.43a	135.76a	1.39a	53.39a	0.86a	5.23a	0.76a	11.15a	343.48a	325.28a	0.086a	1819.53a	125.85a	1.31a	52.05a
	N300+Y	0.92a	5.55a	0.079a	11.74a	335.66bc	323.83ab	0.088b	1822.54ab	126.88ab	1.29b	54.78a	0.83a	5.06a	0.74a	10.92a	325.84b	316.17a	0.084a	1786.67a	117.09b	1.22b	50.83a
Early filling stage	N100+CK	1.55c	5.87d	0.039c	4.85d	456.81c	361.88d	0.095e	1036.46c	143.64c	0.96d	25.91d	1.47d	5.79d	0.34d	5.17d	450.24e	333.92d	0.088d	1087.65d	128.95d	0.90d	22.07e
	N200+CK	1.68b	6.65c	0.045bc	5.83c	486.52b	377.28c	0.103cd	1251.03b	155.73bc	1.07c	28.92c	1.63c	6.27c	0.38c	5.59c	477.91cd	354.38c	0.096c	1174.59c	141.63c	0.99c	28.58c
	N300+CK	1.73b	6.75c	0.044bc	5.95c	475.80b	358.97d	0.098de	1167.03b	151.38bc	1.03c	30.43c	1.59c	6.05cd	0.37c	5.42cd	468.17de	340.03cd	0.093c	1106.27d	134.07d	0.93d	24.94d
	N100+Y	1.77b	7.30b	0.044bc	6.73b	524.52a	392.79b	0.109bc	1248.27b	163.15ab	1.16b	34.17b	1.75b	6.91b	0.41b	6.58b	496.87bc	377.49b	0.104b	1256.76b	152.19b	1.06b	32.31b
	N200+Y	1.94a	7.98a	0.050ab	7.12a	537.46a	411.63a	0.122a	1396.47a	173.51a	1.22a	37.13a	1.85a	7.64a	0.46a	6.94a	525.75a	403.67a	0.115a	1362.09a	169.72a	1.14a	35.85a
	N300+Y	1.98a	7.91a	0.051a	7.28a	525.22a	399.12ab	0.113b	1380.29a	165.35ab	1.16b	37.88a	1.81ab	7.38a	0.45a	6.85ab	520.33ab	396.54a	0.108b	1283.15b	157.94b	1.12a	33.67b
Milk stage	N100+CK	0.37d	1.26d	0.021c	2.64d	117.79c	20.18d	0.067d	481.15d	41.52d	0.17d	15.89c	0.35d	1.31d	0.23d	3.42d	113.06d	24.31e	0.065d	493.17d	42.35d	0.20d	13.77d
	N200+CK	0.46c	1.66c	0.026bc	3.26c	130.83b	30.82c	0.076c	560.38c	48.58c	0.25c	16.56c	0.43c	1.62c	0.26c	3.89c	126.74c	31.38d	0.073c	545.39c	46.88c	0.24c	15.85c
	N300+CK	0.48c	1.80bc	0.028bc	3.54c	129.95b	32.04c	0.069d	539.03c	46.93cd	0.25c	17.03c	0.41c	1.57c	0.24d	3.57d	118.38d	25.47e	0.068d	516.28d	44.27d	0.21d	14.42d
	N100+Y	0.59b	1.92b	0.026b	4.31b	155.06a	38.73b	0.085b	622.59b	57.07b	033a	20.97b	0.56b	1.85b	0.30b	4.36b	139.02b	36.79c	0.079b	603.05b	52.53b	0.29b	20.51b
	N200+Y	0.68a	2.33a	0.031ab	4.65ab	167.65a	49.55a	0.091a	712.55a	64.38a	0.35a	24.25a	0.64a	2.26a	0.33a	4.73a	158.85a	45.32a	0.088a	684.91a	57.96a	0.32a	22.69a
	N300+Y	0.69a	2.44a	0.034a	4.97a	162.60a	46.57a	0.089ab	676.37a	58.75ab	0.27b	25.50a	0.62a	2.18a	0.31b	4.48b	152.37a	42.68b	0.085a	627.56b	54.19b	0.30b	21.18b

*N100+CK, N200+CK, and N300+CK indicate nitrogen applied levels at 100, 200, and 300 kg ha^−1^ under water treatment, respectively; N100+Y, N200+Y, and N300+Y indicate nitrogen applied levels at 100, 200, and 300 kg ha^−1^ under chemical control, respectively. Means within a column for the same growth stage followed by the different letters indicate a significant difference at P < 0.05*.

### P and K Accumulation and Translocation

Changes between the P and K accumulation in maize plants followed similar trends; both P and K increased gradually from the jointing stage to thewe maturing stage (Table 4). Chemical control and N fertilization level exhibited a marked influence on P and K accumulation amount during the maize growing period in both years. At the same N levels, chemical control increased P accumulation amount with an average augment of 4.48, 15.34, 22.07, 23.52, and 24.32% and K accumulation amount with an average augment of 6.30, 14.43, 17.60, 18.94, and 19.55% from the jointing stage to the maturing stage in 2017 and 2018. Under both water and chemical control conditions, P and K accumulation amount increased by increasing the N level from N100 to N300, but there was no significant difference between N200 and N300 treatments in both years. Compared with N100, N200 and N300 treatments increased P and K accumulation amount with an average augment of 22.41 and 24.26%, respectively.

**Table 4:** Effects of chemical control and nitrogen fertilizers on P and K accumulation (kg ha^−1^) during the maize growing period in 2017 and 2018.

**Nutrient**	**Treatment**	**2017**					**2018**				
		**Jointing stage**	**Tasseling stage**	**Early filling stage**	**Milk stage**	**Maturing stage**	**Jointing stage**	**Tasseling stage**	**Early filling stage**	**Milk stage**	**Maturing stage**
P	N100+CK	9.88c	22.02c	26.96c	29.08d	30.31d	9.55c	20.78c	25.81c	27.82c	28.95c
	N200+CK	10.26bc	25.55b	32.52b	36.78bc	39.09bc	10.02bc	23.74b	30.64b	34.35b	36.37b
	N300+CK	10.45b	26.32b	33.57b	37.83b	39.85b	10.38b	24.66b	31.89b	35.77b	37.81b
	N100+Y	10.20b	25.48b	32.78b	36.03c	38.01c	10.04b	23.62b	31.16b	34.29b	36.13b
	N200+Y	10.76a	29.85a	40.08a	45.65a	48.64a	10.61a	27.81a	38.27a	43.43a	46.18a
	N300+Y	10.96a	30.10a	40.37a	45.86a	48.65a	10.68a	28.17a	38.75a	43.77a	46.38a
K	N100+CK	37.0.88c	72.56c	93.49c	105.01d	114.71d	37.49c	70.15c	89.64c	100.87c	109.61c
	N200+CK	39.53bc	81.61b	107.64b	123.89bc	137.60bc	39.36bc	77.42b	101.27b	114.92b	125.88b
	N300+CK	41.21b	85.18b	112.92b	129.75c	143.62c	39.91b	78.79b	103.25b	117.38b	128.53b
	N100+Y	39.95b	82.01b	107.51b	122.60b	134.81b	38.67bc	76.04b	99.21b	112.45b	123.04b
	N200+Y	42.89a	96.47a	130.70a	152.04a	169.10a	42.52a	89.88a	120.96a	139.24a	153.72a
	N300+Y	43.05a	97.22a	133.48a	154.80a	172.73a	43.13a	91.37a	123.52a	141.91a	156.29a

*N100+CK, N200+CK, and N300+CK indicate nitrogen applied levels at 100, 200, and 300 kg ha^−1^ under water treatment, respectively; N100+Y, N200+Y, and N300+Y indicate nitrogen applied levels at 100, 200, and 300 kg ha^−1^ under chemical control, respectively. Means within a column for the same nutrient followed by the different letters indicate a significant difference at P < 0.05*.

Changes in the proportion of P and K accumulation in maize plants during various growth stages seemed to follow similar trends (Table 5). Proportions of P and K accumulation had a higher value at emerging (VE) —jointing (JT) and JT—tasseling (TS) stages and decreased gradually from TS—early-filling (EF) to milk (MK)—maturing (MT) stage. The proportions of P and K accumulation were significantly affected by chemical control and N fertilization level. At the same N levels, chemical control increased the proportions of P and K accumulation at TS-EF, EF-MK, and MK-MT stages, while the proportions decreased at the VE-JT stage and remained relatively constant at the JT-TS stage in 2017 and 2018. Under both water and chemical control conditions, N supply significantly increased the proportions of P and K accumulation. However, there was no significant difference between N200 and N300 treatments, and the highest proportions were obtained under N200 treatment at EF-MK and MK-MT stages in both years.

**Table 5:** Effects of chemical control and nitrogen fertilizers on the proportion of P and K accumulation (%) at different maize growing stages in 2017 and 2018.

**Nutrient**	**Treatment**	**2017**					**2018**				
		**VE-JT**	**JT-TS**	**TS-EF**	**EF-MK**	**MK-MT**	**VE-JT**	**JT-TS**	**TS-EF**	**EF-MK**	**MK-MT**
P	N100+CK	32.60a	40.04a	16.31d	6.98d	4.07d	32.99a	38.79a	17.37d	6.94d	3.90d
	N200+CK	26.25b	39.12a	17.82c	10.91b	5.90b	27.55b	37.72a	18.97c	10.20b	5.55b
	N300+CK	26.23b	39.83a	18.19c	10.69b	5.05c	27.45b	37.77a	19.12c	10.26b	5.40b
	N100+Y	26.85b	40.19a	19.21b	8.55c	5.21c	27.79b	37.59a	20.87b	8.66c	5.09c
	N200+Y	22.12c	39.26a	21.02a	11.46a	6.14a	22.98c	37.25a	22.65a	11.17a	5.96a
	N300+Y	22.54c	39.34a	21.11a	11.28a	5.73b	23.03c	37.71a	22.81a	10.82a	5.63b
K	N100+CK	33.02a	30.23a	18.25d	10.04c	8.46d	34.20a	29.80a	17.78c	10.25c	7.97c
	N200+CK	28.73b	30.58a	18.92cd	11.81b	9.96c	31.27b	30.24a	18.95b	10.84b	8.71b
	N300+CK	28.69b	30.62a	19.31bc	11.72b	9.66c	31.05b	30.25a	19.03b	10.99b	8.68b
	N100+Y	29.63b	31.20a	18.92cd	11.19b	9.06b	31.43b	30.37a	18.83b	10.76b	8.61b
	N200+Y	25.36c	31.69a	20.24ab	12.62a	10.09a	27.66c	30.81a	20.22a	11.89a	9.42a
	N300+Y	25.07c	31.54a	21.12a	12.41a	9.86a	27.60c	30.87a	20.57a	11.77a	9.20a

*VE, emerging stage; JT, jointing stage; TS, tasseling stage; EF, Early filling stage; MK, milk stage; MT, maturing stage. N100+CK, N200+CK, and N300+CK indicate nitrogen applied levels at 100, 200, and 300 kg ha^−1^ under water treatment, respectively; N100+Y, N200+Y, and N300+Y indicate nitrogen applied levels at 100, 200, and 300 kg ha^−1^ under chemical control, respectively. Means within a column for the same nutrient followed by the different letters indicate a significant difference at P < 0.05*.

Chemical control and nitrogen fertilizer significantly influenced the nutrient (P and K) translocation and contribution, including the vegetative organ nutrient content at the silking stage (VCS), the vegetative organ nutrient content at the maturing stage (VCM), and the grain nutrient content at the maturing stage (GCM), the nutrient translocation amount of pre-silking (TAE), the nutrient translocation rate of pre-silking (TRE), the contribution rate of nutrient translocation amount of pre-silking (CTAE), the nutrient accumulation amount of post-silking (AAT), and the contribution rate of nutrient accumulation amount of post-silking (CAAT) (Table 6). At the same N levels, VCS, VCM, GCM, TAE, AAT, and CAAT of P and K in maize plants under chemical control were markedly higher than those under water treatment. In contrast, TRE and CTAE of P and K in maize plants under chemical control were markedly lower than those under water treatment. Under both water and chemical control conditions, VCS, VCM, GCM, and TAE of P and K in maize plants were significantly increased by increasing N levels; however, TRE and CTAE were decreased. While N supply in general significantly increased AAT and CAAT of P and K in maize plants, there is no significant difference between N200 and N300 treatments, and the highest values were obtained under N200 treatment in both years.

**Table 6:** Effects of chemical control and nitrogen fertilizers on maize nutrient (P and K) translocation and contribution during the maize growing period 2017 and 2018.

**Nutrient**	**Treatment**	**2017**								**2018**							
		**VCS (kg ha^**−1**^)**	**VCM (kg ha^**−1**^)**	**GCM (kg ha^**−1**^)**	**TAE (kg ha^**−1**^)**	**TRE (%)**	**CTAE (%)**	**AAT (kg ha^**−1**^)**	**CAAT (%)**	**VCS (kg ha^**−1**^)**	**VCM (kg ha^**−1**^)**	**GCM (kg ha^**−1**^)**	**TAE (kg ha^**−1**^)**	**TRE (%)**	**CTAE (%)**	**AAT (kg ha^**−1**^)**	**CAAT (%)**
P	N100+CK	21.19d	7.54e	22.27c	13.64d	64.40a	61.27a	8.62c	38.73d	20.37c	7.03d	21.02c	13.35d	65.51a	63.49a	7.68c	36.51c
	N200+CK	26.33c	10.26c	28.63b	16.07c	61.04b	56.13c	12.56b	43.87b	24.60b	9.52bc	25.73b	15.09c	61.32b	58.63b	10.65b	41.37b
	N300+CK	27.82b	11.05b	28.80b	16.77b	60.27b	58.24b	12.03b	41.76c	25.49b	9.97b	26.01b	15.52bc	60.90b	59.69b	10.48b	40.31b
	N100+Y	26.08c	9.17d	29.13b	16.91b	64.84a	58.05b	12.22b	41.95c	25.10b	9.07c	26.83b	16.03b	63.87a	59.77b	10.79b	40.23b
	N200+Y	32.16a	13.50a	34.94a	18.66a	58.01c	53.39d	16.29a	46.61a	31.29a	12.92a	33.24a	18.38a	58.73b	55.29c	14.86a	44.71a
	N300+Y	32.24a	13.72a	34.11a	18.52a	57.43c	54.29d	15.59a	45.71a	31.46a	12.89a	33.35a	18.58a	59.04b	55.69c	14.78a	44.31a
K	N100+CK	82.58e	35.07d	78.64e	47.51e	57.53b	60.41a	31.13d	39.59d	79.59d	30.88d	76.73c	48.70c	61.20a	63.48a	28.02d	36.52d
	N200+CK	96.39c	44.87b	94.73d	51.51d	53.44d	54.38c	43.21c	45.62b	89.70bc	39.76b	88.12b	49.94bc	55.67b	56.68c	38.18b	43.32b
	N300+CK	100.76b	44.26b	103.36b	56.50b	56.08c	54.67bc	46.86b	45.33bc	92.03b	40.56b	89.97b	51.47b	55.93b	57.21c	38.50b	42.79b
	N100+Y	91.77d	37.38c	97.43c	54.39c	59.27a	55.82b	43.04c	44.18c	87.11c	35.31c	86.13b	51.80b	59.46a	60.14b	34.33c	39.86c
	N200+Y	111.62a	52.11a	118.51a	59.51a	53.31d	50.22d	59.00a	49.78a	103.24a	46.12a	107.60a	57.12a	55.33b	53.08d	50.48a	46.92a
	N300+Y	112.83a	51.85a	121.03a	60.98a	54.05c	50.38d	60.05a	49.62a	105.96a	46.89a	109.40a	59.08a	55.75b	54.00d	50.33a	46.00a

*VCS, vegetative organ nutrient (P or K) content at silking stage; VCM, vegetative organ nutrient (P or K) content at maturing stage; GCM, grain nutrient (P or K) content at maturing stage; TAE, nutrient (P or K) translocation amount of pre-silking; TRE, nutrient (P or K) translocation rate of pre-silking; CTAE, contribution rate of nutrient (P or K) translocation amount of pre-silking; AAT, nutrient (P or K) accumulation amount of post-silking; and CAAT, contribution rate of nutrient (P or K) accumulation amount of post-silking. N100+CK, N200+CK, and N300+CK indicate nitrogen applied levels at 100, 200, and 300 kg ha^−1^ under water treatment, respectively; N100+Y, N200+Y, and N300+Y indicate nitrogen applied levels at 100, 200, and 300 kg ha^−1^ under Yuhuangjin treatment, respectively. Means within a column for the same nutrient followed by the different letters indicate a significant difference at P < 0.05*.

### RuBPCase and PEPCase Activities in Leaf

**Figure 2:**
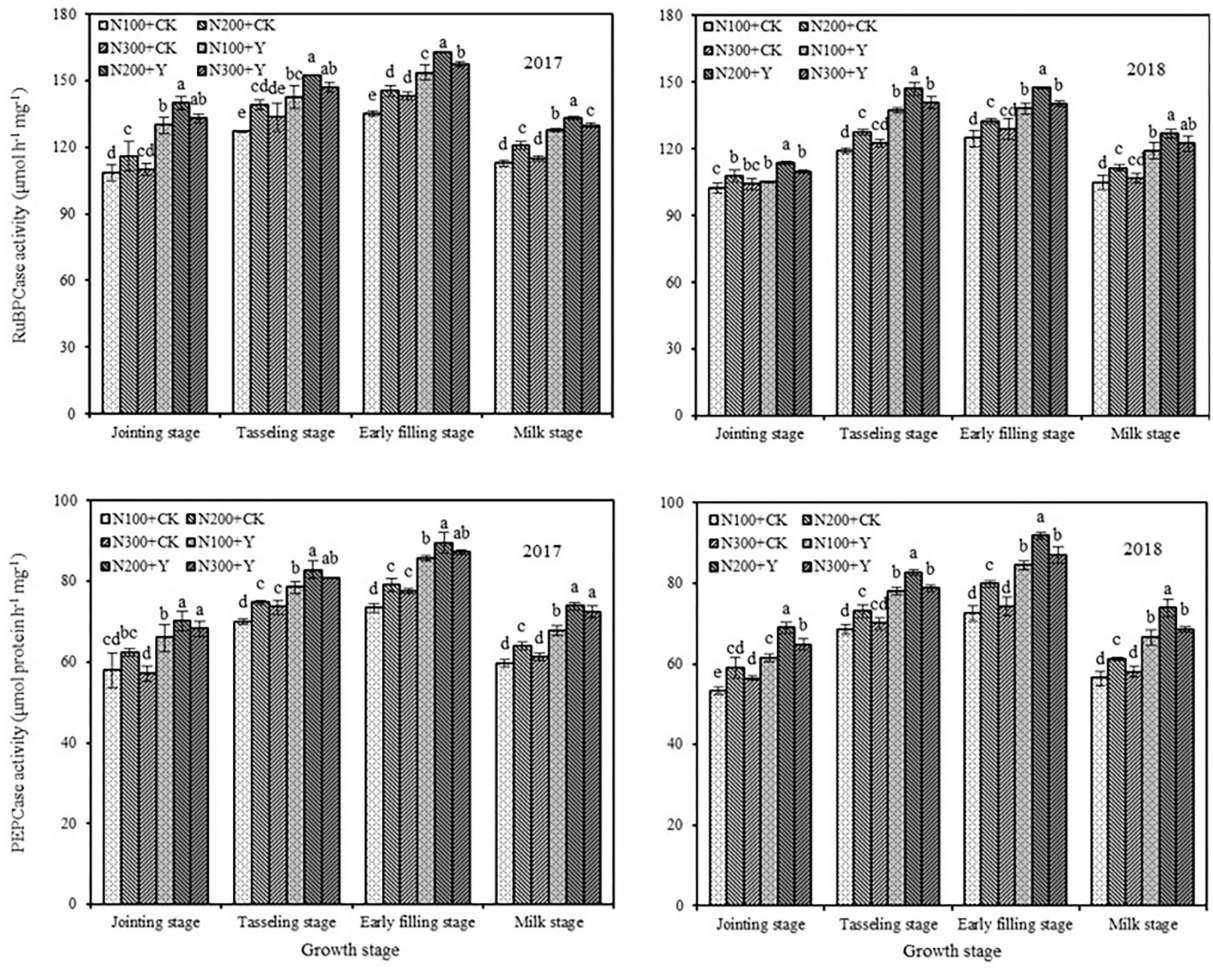
Effects of chemical control and nitrogen fertilizers on RuBPCase and PEPCase activities in ear leaf during the maize growing period in 2017 and 2018. N100+CK, N200+CK, and N300+CK indicate nitrogen applied levels at 100, 200, and 300 kg ha^−1^ under water treatment, respectively; N100+Y, N200+Y, and N300+Y indicate nitrogen applied levels at 100, 200, and 300 kg ha^−1^ under chemical control, respectively. Error bars indicate the value of standard error. Different letters within a growth stage indicate a significant difference at *P* < 0.05.

Chemical control and N fertilization level exhibited a marked influence on RuBPCase activity in leaves during the maize growing period in 2017 and 2018 (Figure 2). At the same N levels, chemical control increased RuBPCase activity with an average augment of 12.45, 12.91, 11.03, and 13.02% from the jointing stage to the milk stage in 2017 and 2018, respectively. Under both water and chemical control conditions, RuBPCase activity increased with an average augment of 6.78% by increasing the N supply level from N100 to N200 in both years, but further increasing the N supply level from N200 to N300 decreased RuBPCase activity at different stages. From the analysis of synthetic effect, RuBPCase activity in maize leaf was optimal in N200 application under chemical control.

A similar trend was also observed for PEPCase activity in maize leaf, and the activity was significantly affected by chemical control and N fertilization levels (Figure 2). At the same N levels, chemical control increased PEPCase activity with an average augment of 15.46, 11.98, 15.13, and 17.43% from the jointing stage to the milk stage in 2017 and 2018, respectively. Under both water and chemical control conditions, PEPCase activity under N200 treatment was higher than those under N100 and N300 treatments, with an average augment of 7.87 and 4.46% at different stages, respectively. From the analysis of synthetic effect, PEPCase activity in maize leaf was optimal in N200 application under chemical control.

### NR and GS Activities in Leaf

**Figure 3:**
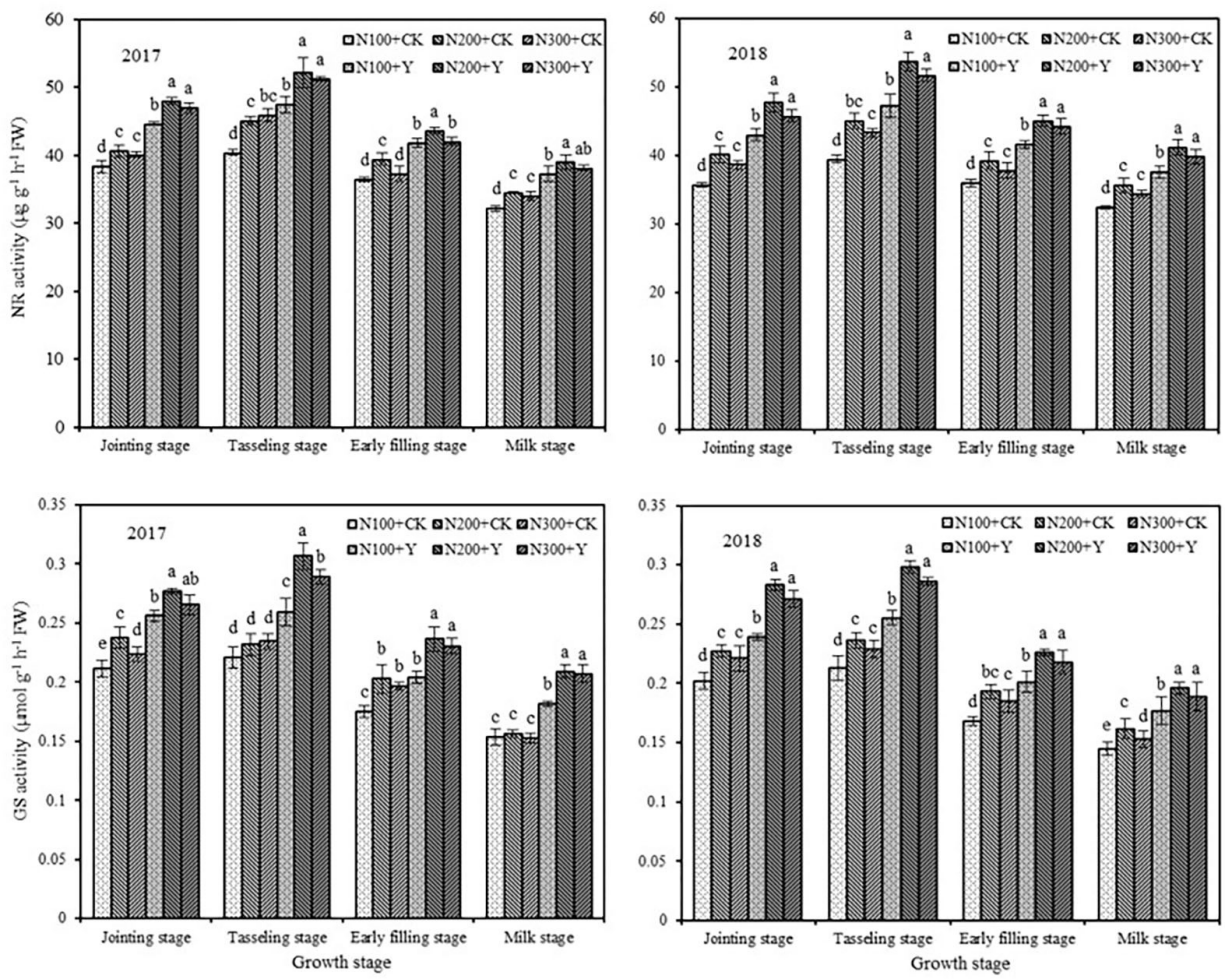
Effects of chemical control and nitrogen fertilizers on NR and GS activities in ear leaf during the maize growing period in 2017 and 2018. N100+CK, N200+CK, and N300+CK indicate nitrogen applied levels at 100, 200, and 300 kg ha^−1^ under water treatment, respectively; N100+Y, N200+Y, and N300+Y indicate nitrogen applied levels at 100, 200, and 300 kg ha^−1^ under chemical control, respectively. Error bars indicate the value of standard error. Different letters within a growth stage indicate a significant difference at *P* < 0.05.

Chemical control and N fertilization level exhibited a marked influence on NR and GS activities in leaves during the maize growing period in 2017 and 2018 (Figure 3). At the same N levels, chemical control increased NR activity with an average augment of 18.23, 17.11, 14.32, and 14.71% and increased GS activity with an average augment of 20.28, 24.12, 17.41, and 25.69% from the jointing stage to the milk stage in both years, respectively. Under water and chemical control conditions, NR and GS activities were significantly increased by increasing the N level from N100 to N200, but further increasing the N supply level from N200 to N300 caused a decrease in NR and GS activities at different stages. From the analysis of synthetical effect, NR and GS activities in maize leaf were optimal in N200 application under chemical control.

### N Metabolism Enzyme Activity in Grain

**Figure 4:**
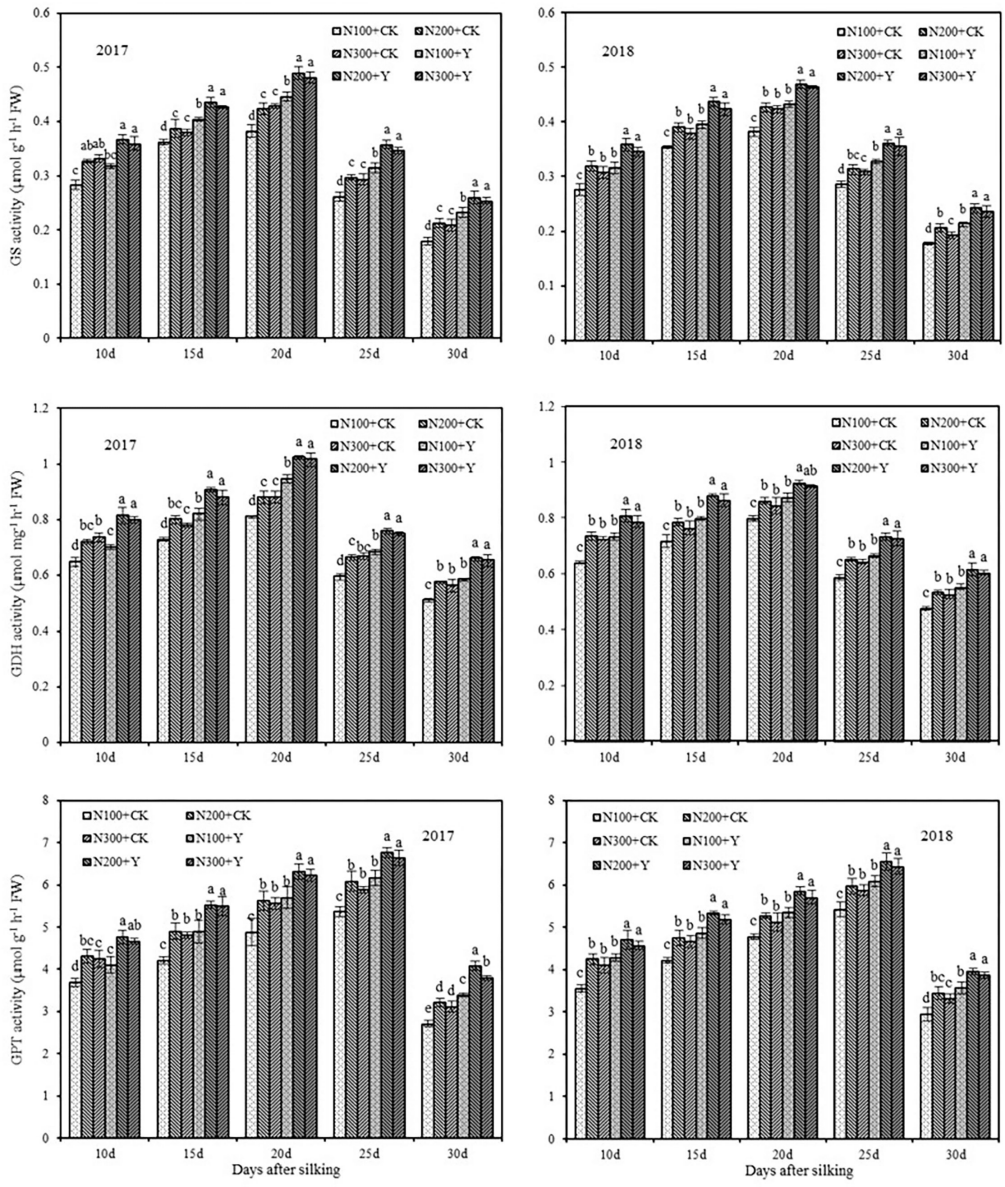
Effects of chemical control and nitrogen fertilizers on GS, GDH, and GPT activities in grain from 10 to 30 days after silking in 2017 and 2018. N100+CK, N200+CK, and N300+CK indicate nitrogen applied levels at 100, 200, and 300 kg ha^−1^ under water treatment, respectively; N100+Y, N200+Y, and N300+Y indicate nitrogen applied levels at 100, 200, and 300 kg ha^−1^ under chemical control, respectively. Error bars indicate the value of standard error. Different letters within a growth stage indicate a significant difference at *P* < 0.05.

Chemical control and N fertilization level exerted a marked effect on grain GS, GDH, and GPT activities from 10 to 30 days after silking in 2017 and 2018 (Figure 4). Of these, GS and GDH activities increased between 10 and 20 days after silking and then decreased until 30 days after silking. However, GPT activity fluctuated with grain growth, which was highest and lowest at 25 and 30 days after silking, respectively. At the same N levels, chemical control increased GS, GDH, and GPT activities with an average augment of 15.22, 12.76, and 14.21% from 10 to 30 days after silking in both years, respectively. Under both water and chemical control conditions, GS, GDH, and GPT activities in grain were significantly increased by increasing the N supply level from N100 to N200 in both years, but further increasing the N supply level from N200 to N300 caused a slight decrease in grain N metabolism enzyme activities. From the analysis of synthetic effect, N metabolism enzyme activities in grain were optimal in N200 application under chemical control.

### Nutrients Concentrations in Grain

At the same N levels, chemical control significantly increased crude protein, lysine, sucrose, and soluble sugar concentrations of maize compared with water treatment in 2017 and 2018 (Table 7). Crude protein and lysine concentrations were significantly increased by increasing the N supply level from N100 to N200, but further increasing the N supply level from N200 to N300 caused a significant decrease in 2017 and a slight decrease in 2018. Similar trends were also observed for sucrose and soluble sugar concentrations of maize grain. Crude fat and starch concentrations were unaffected by chemical control and N fertilization level. The results show that nutrient concentrations in maize grain were optimal in N200 application under chemical control.

**Table 7:** Effects of chemical control and nitrogen fertilizers on grain nutrients concentrations (%) of maize during maize growing period 2017 and 2018.

**Year**	**Treatment**	**Crude protein**	**Crude fat**	**Starch**	**Lysine**	**Sucrose**	**Soluble sugar**
2017	N100+CK	9.53e	5.16a	71.81a	0.43d	1.02e	1.68d
	N200+CK	10.67c	5.20a	73.14a	0.47b	1.11b	1.78b
	N300+CK	10.06d	5.16a	72.56a	0.45c	1.07d	1.72cd
	N100+Y	10.78c	5.14a	71.69a	0.45c	1.09c	1.74bc
	N200+Y	11.78a	5.26a	73.18a	0.49a	1.15a	1.85a
	N300+Y	11.33b	5.22a	72.97a	0.47b	1.12b	1.82a
2018	N100+CK	9.05e	5.21a	71.63a	0.42c	1.02c	1.67c
	N200+CK	10.12cd	5.28a	73.57a	0.45b	1.14b	1.80b
	N300+CK	9.67d	5.23a	72.35a	0.45b	1.09b	1.75bc
	N100+Y	10.29bc	5.24a	72.06a	0.45b	1.10b	1.79b
	N200+Y	11.18a	5.34a	73.94a	0.49a	1.17a	1.93a
	N300+Y	10.74ab	5.29a	72.68a	0.48a	1.11ab	1.88ab

*N100+CK, N200+CK, and N300+CK indicate nitrogen applied levels at 100, 200, and 300 kg ha^−1^ under water treatment, respectively; N100+Y, N200+Y, and N300+Y indicate nitrogen applied levels at 100, 200, and 300 kg ha^−1^ under chemical control, respectively. Means within a column for the same year followed by the different letters indicate a significant difference at P < 0.05*.

### Yield and Yield Components

Chemical control and N fertilization level exhibited a marked influence on yield and yield components of maize in 2017 and 2018 (Table 8). Chemical control significantly increased the number of grains per ear and 1,000-grain weight compared with maize under water treatment in 2017 and 2018. Grain number per ear and 1,000-grain weight significantly increased by increasing the N supply level from N100 to N200, but further increasing the N supply level from N200 to N300 caused a slight decrease in 2017 and 2018. The highest grain yields were obtained from the N200 application under chemical control in 2017 and 2018.

**Table 8:** Effects of chemical control and nitrogen fertilizers on yield and yield components of maize during the maize growing period 2017 and 2018.

**Year**	**Treatment**	**Ears number per ha**	**Grains number per ear**	**1,000-grain weight (g)**	**Yield (kg ha^**−1**^)**
2017	N100+CK	81,078a	541c	332b	10511c
	N200+CK	81,654a	568b	327b	11548b
	N300+CK	81,782a	560b	316c	11053bc
	N100+Y	81,657a	571b	340ab	11427b
	N200+Y	81,683a	591a	351a	12646a
	N300+Y	82,150a	570b	339ab	11921b
2018	N100+CK	80,325a	531c	294c	9840bc
	N200+CK	80,793a	550bc	298bc	10430b
	N300+CK	78,685b	533c	298bc	9204c
	N100+Y	81,052a	556abc	306bc	9990bc
	N200+Y	81,184a	581a	327a	11704a
	N300+Y	81,167a	566ab	314ab	10732ab

*N100+CK, N200+CK, and N300+CK indicate nitrogen applied levels at 100, 200, and 300 kg ha^−1^ under water treatment, respectively; N100+Y, N200+Y, and N300+Y indicate nitrogen applied levels at 100, 200, and 300 kg ha^−1^ under chemical control, respectively. Means within a column for the same year followed by the different letters indicate a significant difference at P < 0.05*.

### Correlation Analysis

**Figure 5:**
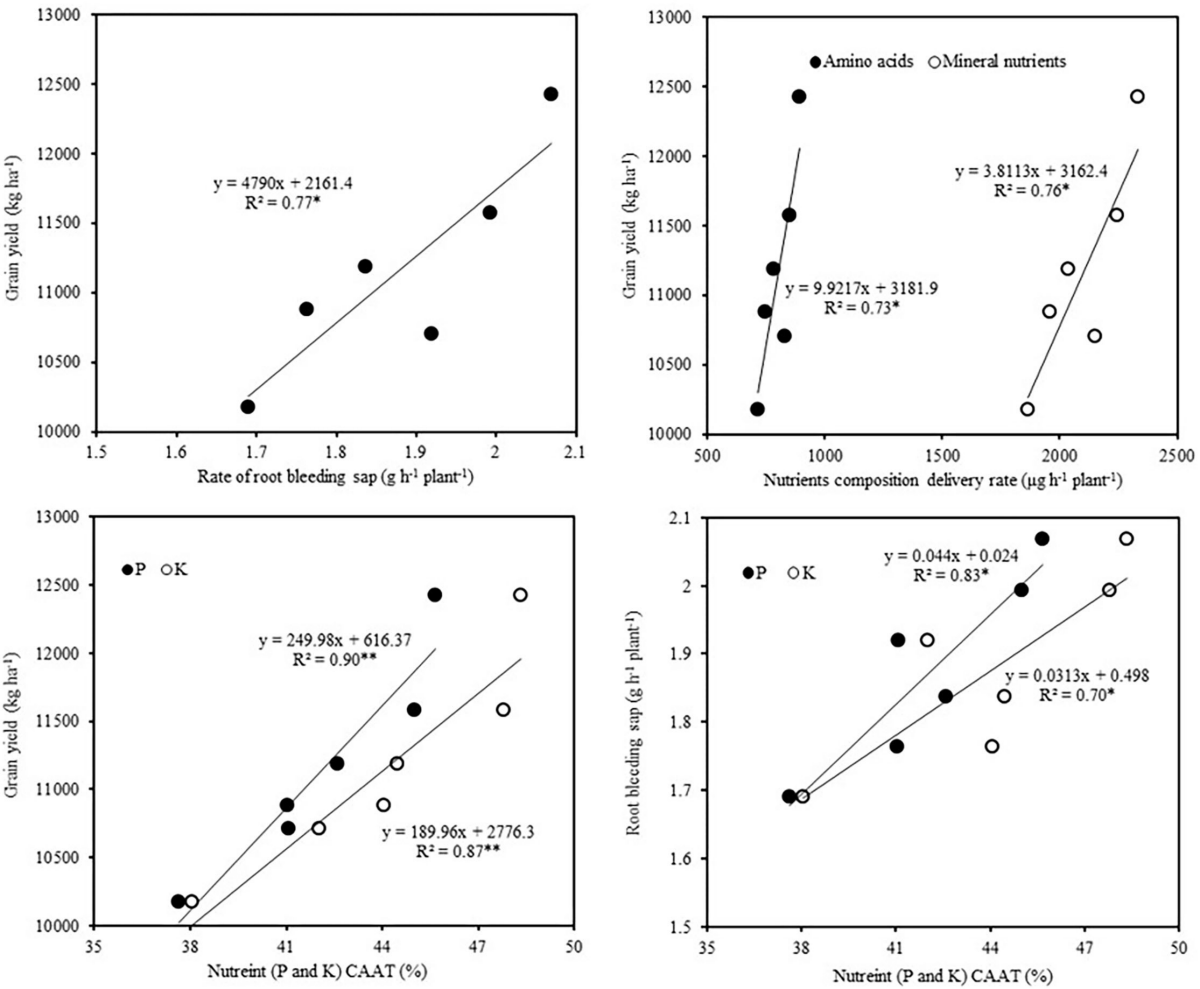
Correlation analysis of root-bleeding sap, nutrient contribution, and grain yield (values are the average in both years). CAAT, contribution rate of nutrient (P or K) accumulation amount post-silking.

As shown in Figure 5, correlation analysis indicated that grain yield was positively correlated with the rate of root-bleeding sap, the delivery rate of amino acids and mineral nutrients in the bleeding sap, and CAAT of P and K. Besides, the CAAT of P and K were positively correlated with the rate of root-bleeding sap.

## Discussion

The root system is an essential source for uptake of water and nutrients, and its physiological activity is closely correlated to the development of the plant's parts above ground and the yield formation of crops (Yang et al., 2004; Fan et al., 2021). Root-bleeding sap reflects the capacity of roots to uptake water and nutrients, and it represents the physiological activity of the root system (Ansari et al., 2004; Wang P. et al., 2019). It has been found that root growth is closely associated with root-bleeding sap rate. The reduction of root quality in high-density planting seriously affects yield formation (Yu et al., 2019; Liu Z. et al., 2021). A balanced application of nitrogen can enhance root activity by supplying nutrients to form a robust root system (Wang H. et al., 2019). Equally, chemical control can optimize root morphological construction and improve the absorption ability of the root system (Lin et al., 2019).

In this study, N200 application in combination with chemical control significantly enhanced the rate of root-bleeding sap to enhance the strength of root activity. The nutrient concentrations in root-bleeding sap are closely associated with the absorption and transformation capacity of the root system, and its variation reflects the interaction intensity of nutrients in the aboveground and underground plant parts (Nishanth and Biswas, 2008). The xylem sap can transport nutrients upward to the aboveground tissues. The nutrient concentrations in root-bleeding sap are generally recognized as indicators of the plant's nutrient status (Ansari et al., 2004). Amino acids are essential for maintaining plant growth and, when contained in root-bleeding sap, promote root growth (Zheng et al., 2020). Mineral nutrient concentration is considered a primary factor for plant growth and grain yield. The delivery rate of mineral nutrients primarily depends on the root physiological activity and the nutrient concentrations across the root zone (Liang et al., 2020). High-density planting reduces root physiological activity and intensifies the depletion of nutrients in the root zone, resulting in the reduction of free amino acids and mineral nutrient concentrations (Yu et al., 2012; Liang et al., 2020). The content of free amino acids varied significantly with different nitrogen nutrient levels. It is believed that the delivery rate of free amino acids in root-bleeding sap increases with an increasing rate of nitrogen application (Li et al., 2009). In the present study, we found that N200 application combined with chemical control increased the delivery rate of amino acids and mineral nutrients in root-bleeding sap. The proper cultivation measure can improve the capacity of roots to absorb, synthesize, and transport carbohydrates, auxin, and other substances, thereby promoting root activity and root growth (Wang H. et al., 2019). The increase in root activity and its capacity for water and nutrients could lay the foundation for the increase in maize yield under high-density planting.

Nutrient absorption and accumulation are the basis of crop yield formation, and it directly affects the growth process of crops (Wu et al., 2018; Gorlach et al., 2021). Nutrient absorption in maize increases with plant growth. Sufficient nutrient supply during the growth period is the key to obtaining a high maize yield (Ray et al., 2020). Phosphorus and potassium are essential macronutrient elements for maize growth, which play an important role in the yield potential (Wu et al., 2015; Zhan et al., 2016). Nitrogen fertilizer is recognized to be an important factor affecting nutrient accumulation and transportation in addition to chemical control, which also impacts plant nutrient absorption capability (Van Oosten et al., 2019; Ray et al., 2020). In the present study, chemical control increased P and K accumulation amounts at different N levels. P and K accumulation amount increased with increasing level of N application, but the differences between N200 and N300 treatments were not significant. The nutrient accumulation by plants during different growth stages may impact crop yield. It is believed that the high nutrient absorption of N, P, and K in the middle growth stage of crops can promote pre-anthesis non-structural carbohydrate (NSC) reserves in the stem and accordingly enhance grain sink strength during grain filling (Fu et al., 2011; Li W. H. et al., 2012). Liu et al. (2019) considered that the P and K nutrient absorption in the late growth stage played an important role in improving maize production. In the present study, chemical control and nitrogen fertilizer greatly influenced the proportion of P and K accumulation during different growth stages in maize plants. Similarly, chemical control significantly increased the proportion of P and K accumulation during different growth stages except for the VE-JT and JT-TS stages. The proportion of P and K accumulation after the tasseling stage was obviously increased with increasing levels of N application. Chemical control and nitrogen fertilizer application substantially improved the CAAT of P and K in maize plants, and the highest CAAT of P and K were recorded under N200 application in combination with chemical control. The above results indicate that chemical control and nitrogen fertilizers can improve nutrient accumulation in maize after tasseling and increase the transfer of nutrients from vegetative organs to grains, consequently providing a material basis for yield formation. This result is similar to the study by Ray et al. (2020), which found that appropriate nutrient accumulation and translocation after silking created good conditions for maintaining the supply of nutrients to the grains, resulting in increased yields.

Carbon and nitrogen metabolism determines the level of crop production and function to provide the main energy and basic nutrients for plants (Cui et al., 2019). RuBPCase, PEPCase, NR, and GS are key enzymes involved in carbon and nitrogen metabolism in plants. In the present study, chemical control combined with N200 treatment increased RuBPCase, PEPCase, NR, and GS activities, leading to more assimilate accumulation and higher grain yield (Cheng et al., 2019; Yang et al., 2020). The plants maintained a high carbon and nitrogen metabolism and nutrient accumulation, which was the basis for assimilate accumulation in the grains. Main enzymes such as GS, GPT, and GDH are involved in the nitrogen metabolism in grains, and their activities directly affect the synthesis of amino acids and protein in grains (Wang et al., 2016). The N200 application, in combination with chemical control, significantly increased amino acid and protein content in grains, which in turn increased GS, GPT, and GDH activities. Chemical control and N200 treatments also increased the sucrose and soluble sugar contents of grains. This may be due to its association with higher sucrose metabolism and key enzyme activities (Kaur et al., 2018).

Increasing planting density is one of the important practices to increase maize yield per unit area in agricultural production (Tang et al., 2018). However, high-density planting intensifies the competition for light, nutrients, moisture, and space between maize plants, which restricts the growth of shoot and root systems, resulting in reduced crop yield (Rossini et al., 2011). The root system is the crop organ responsible for the uptake of nutrients, and a higher root activity enhances the nutrient absorption capacity in the root system (Yang et al., 2004). In the present study, the rate of root-bleeding sap was positively correlated with the contribution rate of nutrient (P or K) accumulation amount post-silking. It showed that the enhancement of root activity might be an effective method to develop the absorption and utilization capacity of P and K. Maintaining a relatively high level of root activity is an important approach to improving maize production. Niu et al. (2020) showed that increased root activity ensured the availability of soil nutrients and boosted photosynthetic capacity and biomass production, which are critical for grain filling and yield formation. In the present study, the grain yield was positively correlated with the rate of root-bleeding sap, the delivery rate of amino acids and mineral nutrients in bleeding sap, and the CAAT of P and K. It further confirmed that maintaining higher root activity and absorption and utilization capacity of P and K are the important approaches to obtaining high yields. Establishing a well-developed root system and efficient plant population can promote photosynthate production and nutrient accumulation and improve phosphorus and potassium distribution ratios after silking. Excessive nutrient transfer after silking usually affects the photosynthesis in leaves at a later growth stage, resulting in acceleration of leaf and root senescence and limiting yield improvement. However, deficient nutrient transfer after silking is harmful to grain filling, making it difficult to achieve a high yield. Therefore, appropriate cultivation methods can coordinate nutrient transfer and nutrient accumulation after silking and optimize the source-sink relationship, which plays an important role in improving yield. Our study on maize cultivation in Northeast China indicated that N200 combined with chemical control could optimize P and K absorption and translocation in the later growth stage by increasing root activity, thereby improving grain yield and quality.

## Conclusion

**Figure 6:**
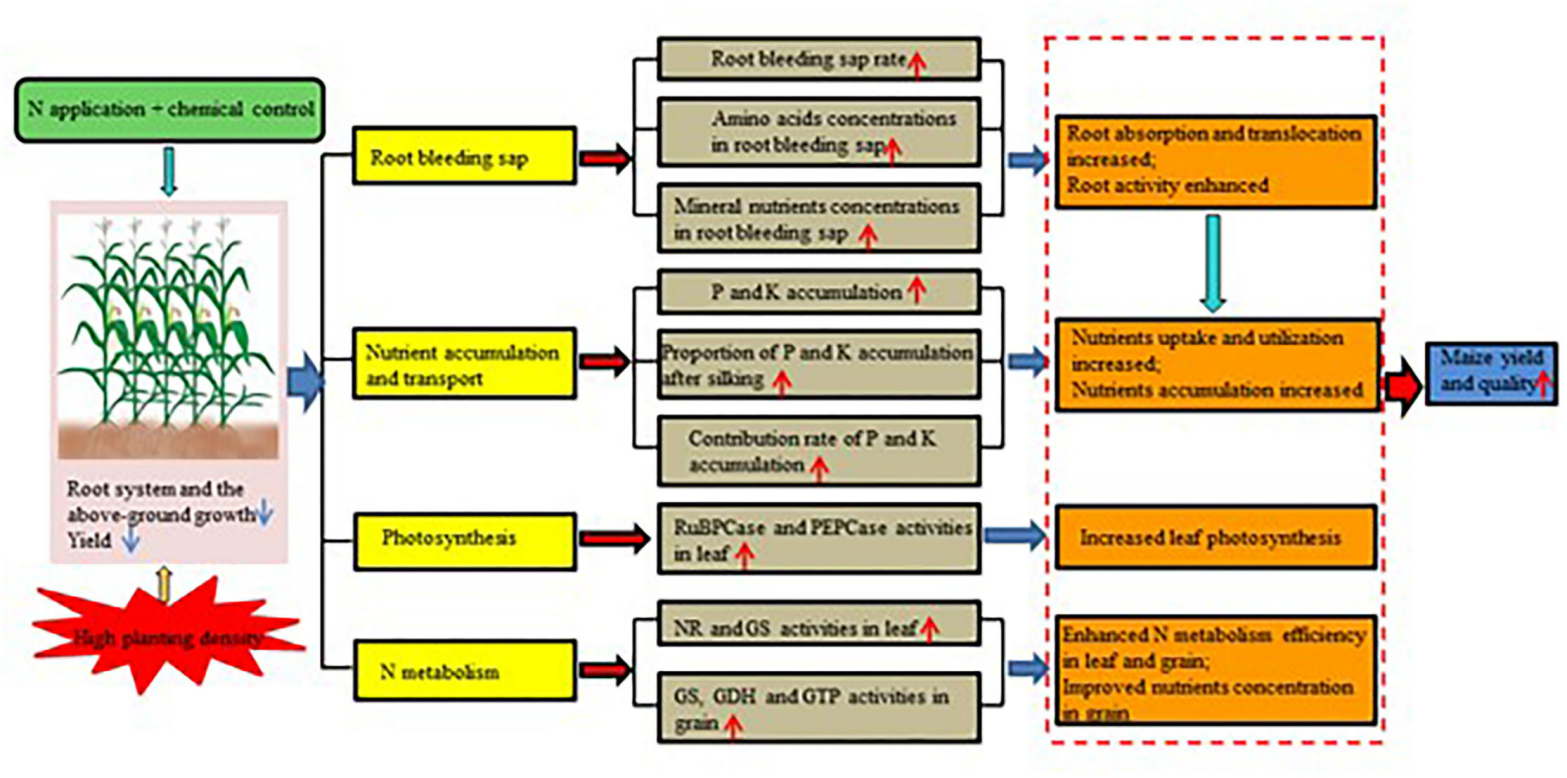
The schematic representation of nitrogen fertilizers and chemical control regulated maize yield. The red arrows (↑) and the blue arrows (↓) represent the positive and passive roles of treatment, respectively.

N200 application in combination with chemical control significantly increased the root-bleeding sap rate, amino acid delivery rate, and mineral nutrient delivery rate. It promoted the accumulation and translocation of P and K nutrients after the tasseling stage, and as a result, it provided a material basis for yield formation. Moreover, N200 combined with chemical control obviously enhanced enzyme activities of carbon and nitrogen metabolism in leaves, increased nitrogen metabolism enzyme activities in grains during the early and middle grain filling stage, and improved amino acid and protein content in grains, thereby increasing the grain yield and quality of maize in high-density planting. The schematic representation indicates that nitrogen fertilizers and chemical control increased the grain yield and quality by optimizing root-bleeding sap, nutrient accumulation and transport, photosynthesis, and N metabolism in maize under high-density planting (Figure 6). Therefore, attention should be paid to promoting nitrogen fertilizer and chemical control management in high-density planting of maize in future agricultural production in Northeast China as it plays a crucial role in improving maize yield and quality.

## Data Availability Statement

The original contributions presented in the study are included in the article/supplementary material, further inquiries can be directed to the corresponding author/s.

## Author Contributions

XL and LZ collected and analyzed the samples and wrote the manuscript. YY, CQ, and CoL contributed to the writing and editing of the manuscript. SW, CaL, and WG contributed to the design of the work and analysis and revised the manuscript. All authors read and approved the article.

## Funding

This study was financially supported by the National Key Research and Development Program of China (2016YFD0300103) and the National Modern Agriculture Industry Technology System (CARS-02-12).

## Conflict of Interest

The authors declare that the research was conducted in the absence of any commercial or financial relationships that could be construed as a potential conflict of interest.

## Publisher's Note

All claims expressed in this article are solely those of the authors and do not necessarily represent those of their affiliated organizations, or those of the publisher, the editors and the reviewers. Any product that may be evaluated in this article, or claim that may be made by its manufacturer, is not guaranteed or endorsed by the publisher.
